# Supplementary material for: Comparative genomics provides new insights into the diversity, physiology, and sexuality of the only industrially exploited tremellomycete: Phaffia rhodozyma
Source: BMC Genomics. 2016 Nov 9;17:901. doi: 10.1186/s12864-016-3244-7 (PMC5103461; doi:10.1186/s12864-016-3244-7)
Supplement: Additional file 6: — List of orphan genes with links to PFAM (related to Additional file 1: Table S1). (ZIP 1428 kb) [file 12864_2016_3244_MOESM6_ESM.zip › PFAM_UNIQUE/G02859_P.html]

#                                                                            --- full sequence --- -------------- this domain -------------   hmm coord   ali coord   env coord
  
# target name        accession   tlen query name           accession   qlen   E-value  score  bias   #  of  c-Evalue  i-Evalue  score  bias  from    to  from    to  from    to  acc description of target
  
#------------------- ---------- ----- -------------------- ---------- ----- --------- ------ ----- --- --- --------- --------- ------ ----- ----- ----- ----- ----- ----- ----- ---- ---------------------
  
G02859\_P             -            138 DUF2484              PF10658.4     77     0.098   11.5   0.1   1   2   1.6e-05     0.098   11.5   0.1     3    32     6    35     1    38 0.90 - Protein of unknown function (DUF2484)  
G02859\_P             -            138 DUF2484              PF10658.4     77     0.098   11.5   0.1   2   2     0.024   1.5e+02    1.3   0.8    32    57    62    87    58    89 0.73 - Protein of unknown function (DUF2484)  
G02859\_P             -            138 DUF4448              PF14610.1    189    0.0018   16.4   0.0   1   1   3.1e-06    0.0021   16.3   0.0   124   188    29    96     9    97 0.72 - Protein of unknown function (DUF4448)  
G02859\_P             -            138 Herpes\_gE            PF02480.11   439     0.016   12.1   0.0   1   1   3.3e-05     0.018   12.0   0.0   327   385    37    96     9   106 0.51 - Alphaherpesvirus glycoprotein E  
G02859\_P             -            138 Indigoidine\_A        PF04227.7    293     0.078   10.8   2.3   1   1   5.2e-05      0.15    9.9   2.3    47   122     9    82     2    99 0.72 - Indigoidine synthase A like protein  
G02859\_P             -            138 Podoplanin           PF05808.6    162     0.029   12.6   0.1   1   1   1.4e-05     0.043   12.1   0.1   127   155    62    89    30    94 0.78 - Podoplanin  
G02859\_P             -            138 Tad                  PF13400.1     48       1.1    8.1   6.4   1   2     0.015        89    1.9   0.5     8    26     3    22     1    24 0.72 - Putative Flp pilus-assembly TadE/G-like  
G02859\_P             -            138 Tad                  PF13400.1     48       1.1    8.1   6.4   2   2   8.1e-05      0.49    9.2   0.8     3    32    62    90    60    95 0.57 - Putative Flp pilus-assembly TadE/G-like
